# Disentangling pectic homogalacturonan and rhamnogalacturonan-I polysaccharides: Evidence for sub-populations in fruit parenchyma systems

**DOI:** 10.1016/j.foodchem.2017.11.025

**Published:** 2018-04-25

**Authors:** Valérie Cornuault, Sara Posé, J. Paul Knox

**Affiliations:** Centre for Plant Sciences, Faculty of Biological Sciences, University of Leeds, Leeds LS2 9JT, United Kingdom

**Keywords:** AGP, arabinogalactan-protein, AEC, anion-exchange chromatography, CWM, cell wall material, ELISA, enzyme-linked immunosorbent assay, EDC, epitope detection chromatography, GalA, galacturonic acid, GX, glucuronoxylan, HG, homogalacturonan, mAb, monoclonal antibody, NS, neutral sugars, PBS, phosphate-buffered saline, RG-I, rhamnogalacturonan-I, RG-II, rhamnogalacturonan-II, Rha, rhamnose, RT, room temperature, SEC, size exclusion chromatography, XG, xyloglucan, Pectic polysaccharides, Fruits, Cell wall, Homogalacturonan, Rhamnogalacturonan-I, Heteroxylan, Solanaceae, Rosaceae

## Abstract

The matrix polysaccharides of plant cell walls are diverse and variable sets of polymers influencing cell wall, tissue and organ properties. Focusing on the relatively simple parenchyma tissues of four fruits – tomato, aubergine, strawberry and apple – we have dissected cell wall matrix polysaccharide contents using sequential solubilisation and antibody-based approaches with a focus on pectic homogalacturonan (HG) and rhamnogalacturonan-I (RG-I). Epitope detection in association with anion-exchange chromatography analysis indicates that in all cases solubilized polymers include spectra of HG molecules with unesterified regions that are separable from methylesterified HG domains. In highly soluble fractions, RG-I domains exist in both HG-associated and non-HG-associated forms. Soluble xyloglucan and pectin-associated xyloglucan components were detected in all fruits. Aubergine glycans contain abundant heteroxylan epitopes, some of which are associated with both pectin and xyloglucan. These profiles of polysaccharide heterogeneity provide a basis for future studies of more complex cell and tissue systems.

## Highlights

• Dissection of cell wall polysaccharides from four fruit parenchyma systems.
• HG molecules with unesterified regions separable from methyl esterified HG.
• RG-I domains exist in both HG-associated and non-HG-associated forms.
• Soluble xyloglucan and pectin-associated xyloglucan detected in all fruits.
• Detection of pectin-xyloglucan-xylan complexes in aubergine parenchyma.

## 1 Introduction

In fruits and vegetables, texture is one of the main traits to determine market quality and consumer acceptance. Texture is better defined as a group of textural properties (firmness, juiciness, crispness, chewingness, adhesiveness, …) with firmness being one of the most important parameters. Firmness is a complex trait which depends on tissue anatomy, cell wall thickness and strength, adhesion between adjacent cells, cell turgor and the presence of specialised cells (eg: vascular bundles), most of which contain thickened walls (Toivonen & Brummell, 2008; and cites therein). During fruit ripening texture is substantially modified leading to fruit softening which determines postharvest shelf life and concomitant economical losses due to oversoftening. Although loss of cell turgor is important in some fruits, cell wall disassembly during fruit ripening is considered the main factor causing softening (Toivonen & Brummell, 2008; and cites therein). The present work is focused on the influence of cell walls on the mechanical and adhesive properties of cells of parenchyma systems, and its relevance on textural properties of fruits and vegetables.

Plant cell walls are highly complex fibrous composites formed by the associations of diverse hetero-polysaccharides with compositions dependent on cell or organ type, developmental stage and taxonomic grouping. Primary cell walls comprise load-bearing cellulose microfibrils and sets of matrix polysaccharides including pectins (Burton, Gidley, & Fincher, 2010). Pectins are a complex group of polysaccharides generally defined by their galacturonic acid content. Four principal, structurally-distinct, polysaccharides are grouped as pectin: homogalacturonan (HG), rhamnogalacturonan-I (RG-I), rhamnogalacturonan-II (RG-II) and xylogalacturonan and these are interlinked in large pectic macromolecules (Burton et al., 2010). Other matrix components include non-cellulosic, non-pectic polysaccharides such as xyloglucans, heteroxylans and heteromannans. How the different components of cell walls link or associate with each other to generate spatially-defined and coherent materials is still unclear (Burton et al., 2010, Park and Cosgrove, 2015).

To-date, functional studies about the role of HG in the mechanical properties of fruits includes tomato (Schuch et al., 1991, Uluisik et al., 2016), strawberry (Quesada et al., 2009, Santiago-Doménech et al., 2008) and apple (Atkinson et al., 2012), where higher and more complex pectin contents correlate with firmer fruits. Another pectic domain of particular interest in relation to plant cell mechanical properties is pectic RG-I which is characterised after its enzymatic release from pectin preparations. RG-I has a backbone of -4)-α-D-Gal*p*A-(1 → 2)-α-L-Rha*p*-(1- and side branches composed mainly of neutral sugars (NS), arabinan and galactan motifs, and are of highly variable structure and composition. Neutral sugar loss is a general feature of fruit ripening, but the relevance of Gal and/or Ara varies among species: loss of pectic galactans occurs early in tomato and apple, while arabinan depolymerization is the main feature in the case of strawberry (Redgwell, Fischer, Kendal, & MacRae, 1997). Several lines of evidence suggest that galactan-rich forms of RG-I contribute to the firmness of multicellular plant tissues (McCartney, Ormerod, Gidley, & Knox, 2000) while arabinans have been associated with elasticity in guard cells (Jones, Milne, Ashford, & McQueen-Mason, 2003). Specifically, reduced β-galactosidase activity of transgenic tomato and strawberry fruits results in firmer fruits than control lines (Paniagua et al., 2016, Smith et al., 2002). In apple, branched arabinan loss has been correlated with fruit softening during postharvest storage (Peña & Carpita, 2004). In the case of aubergine, pectin content (Esteban, Lopez-Andreu, Martin-Cabrejas & Molla, 1993) and cell wall yield increase, while firmness decreases, during fruit ontogeny (Zaro, Keunchkarian, Chaves, Vicente, & Concellón, 2014). Additionally, arabinan- and galactan-rich side chains have high mobility and likely water-holding properties (Ha, Viëtor, Jardine, Apperley, & Jarvis, 2005). Both side chain motifs can be present in parallel in many cell walls (Redgwell et al., 1997) which render any definitive conclusion on the roles of RG-I side chains in the generation of cell wall mechanical properties difficult. Understanding RG-I heterogeneity therefore remains an important goal of plant cell wall biology. Additionally, understanding the basis and the regulation of RG-I links to pectic HG and potentially to other cell wall matrix glycans is a critical area of study for understanding cell wall functions and associated impacts on fruit and vegetable texture.

Monoclonal antibodies (mAbs) are very useful tools for the study of plant cell walls as they specifically recognize, within complex molecular mixtures, oligosaccharide features – or epitopes – contained within polysaccharides of interest. The Epitope Detection Chromatography (EDC) methodology, uses glycan epitopes as markers to provide information on polysaccharide heterogeneity and can also indicate potential links between cell wall polymers (Cornuault et al., 2014, Cornuault et al., 2015).

Questions around the pectic cell wall matrix molecules include how HG and RG-I domains are arranged together in larger pectic polymers as well as the range of potential links between HG/RG-I domains and other cell wall polymer such as xyloglucan, xylan, arabinogalactan-proteins and cellulose. Several reports demonstrate or indicate potential links between various characterized polysaccharide domains (Cornuault et al., 2014, Femenia et al., 1999, Lin et al., 2016, Tan et al., 2013, Wang and Hong, 2016, Zykwinska et al., 2005). However, the exact nature of these inter-molecular linkages remains unknown. Using sets of mAbs and EDC methods we have analysed cell wall matrix components differentially and sequentially solubilised from the parenchyma-enriched systems of a range of fruits with a focus on obtaining information on HG and RG-I and potential links to other matrix polysaccharides.

## 2 Materials and methods

### 2.1 Plant materials and their processing for cell wall analyses

Tomato (*Solanum lycopersicum* L.), apple (*Malus domestica* Borkh. cv. Granny Smith) and aubergine (*Solanum melongena* L.) were purchased locally and processed at the point of consumer use. Strawberries (*Fragaria* × *ananassa* Duchesne) were grown with a 16:8 h light:dark cycle at 25 °C and harvested when ripe.

Selected fruits were peeled, sliced and lyophilized. Freeze-dried materials were milled with a coffee grinder and 0.5 g dry weight aliquots were extracted with 5 ml of phenol:water solution (4:1, w/v) to prevent cell wall degradation by endogenous enzymes. Mixtures were incubated for 2 h at RT and then homogenized in a Polytron homogenizer in three bursts of 30 s. Samples were then centrifuged for 20 min at 4000*g* and supernatants were decanted. Pellets were washed, to remove residual phenol, with 5 ml of 50 mM sodium acetate buffer pH 4, mixed and centrifuged as previously described, and the resulting supernatant was pooled with the previous one as the phenol extract fraction. Pellets were then sequentially washed with 70% (v/v) aqueous ethanol, 90% (v/v) aqueous ethanol, chloroform:methanol (1:1) and finally acetone to obtain an alcohol-insoluble residue (AIR). This AIR was de-starched by α-amylase treatment in Tris–maleate buffer (10 mM) to obtain the final cell wall material (CWM). The CWMs were precipitated in 75% ethanol at −20 °C for 28 h. The supernatants were discarded. The details concerning the yields of material extracted from the parenchyma tissues of the four fruits can be found elsewhere (Table 1, Cornuault, Posé, & Knox, 2017b). Afterwards, CWMs were extracted by a series of solvents to sequentially solubilize constituent polysaccharides as previously described by Santiago-Doménech et al. 2008. Briefly, 5 mg of the CWM fractions were sequentially extracted with 1 ml of: deionized water; 0.05 M *trans*-1,2-diaminocyclohexane-*N*,*N*,*N′N′*-tetraacetic acid (CDTA) in 0.05 M sodium acetate buffer, pH 6; 0.1 M Na_2_CO_3_ containing 0.1% (w/v) NaBH_4_; and 4 M KOH containing 0.1% (w/v) NaBH_4_. Before dialysis, the alkaline fractions were neutralized using acetic acid. All solubilized fractions were dialysed against distilled water using 1.5 ml tubes for small volume samples. Extracts were aliquoted and frozen at −20 °C until use.

### 2.2 Monoclonal antibodies

Rat mAbs used in this study included JIM7 methylesterified HG (Clausen, Willats, & Knox, 2003), LM19 unesterified HG, LM18 partially methylesterified HG (Verhertbruggen, Marcus, Haeger, Ordaz-Ortiz, & Knox, 2009), LM5 galactan (Andersen et al., 2016), LM13 linear arabinan (Verhertbruggen et al., 2009), LM15 xyloglucan (Marcus et al., 2008), LM25 xyloglucan (Pedersen et al., 2012), LM11 xylan (McCartney, Marcus, & Knox, 2005) and LM28 glucuronoxylan (Cornuault et al., 2015). A new rat mAb, designated LM6-M, directed to α-1,5-arabinan was used to detect arabinan side chains of RG-I. LM6-M is a newly developed monoclonal antibody, of the IgM immunoglobulin class, targeting arabinan. It was obtained after the immunization of rats with sugar-beet RG-I oligosaccharides and LM6-M binds preferentially to six 1,5-arabinosyl residues (Cornuault et al., 2017a). A mouse mAb, INRA-RU1 binding to the RG-I backbone (Ralet, Tranquet, Poulain, Moïse, & Guillon, 2010), was also used.

### 2.3 Epitope detection chromatography (EDC)

Cell wall extracts were analysed by EDC and anion-exchange chromatography (AEC) was performed using a 1 ml HiTrap ANX FF column (GE Healthcare, 17-5162-01). The EDC analysis of the phenol extracts was performed using 5 µl (equivalent to 25 µg phenol extract d.w.) of sample diluted in 2.5 ml of 20 mM sodium acetate buffer pH 4.5. For the sequentially solubilized fractions (water, CDTA, Na_2_CO_3_ and KOH), the EDC was performed using an equal 5 µl volume of the dialysed fractions, also diluted in 2.5 ml of 20 mM sodium acetate buffer pH 4.5. Polysaccharides were eluted, collected and analysed using ELISA as described (Cornuault et al., 2014). The salt elution gradient used was adapted from Cornuault et al., 2015 in which samples were eluted at a flow rate of 1 ml/min using a two-step gradient starting with 20 mM sodium acetate buffer pH 4.5 for 13 ml, then a linear gradient of 0–50% 0.6 M NaCl in 50 mM sodium acetate buffer pH 4.5 over 24 ml, followed by a second gradient from 50-100% 0.6 M NaCl for another 7 ml, and 4 further ml with the salt gradient at maximum (50 mM sodium acetate buffer pH 4.5, 0.6 M NaCl). In total, 48 (1 ml) fractions were collected. In all cases the EDC profiles shown are the averages of at least two independent assays. In some cases samples were alkali-treated that would de-esterify HG prior to EDC analysis. De-esterification of HG was performed using a 2.5 h treatment of samples with 0.5 M Na_2_CO_3_ at RT. The treated sample (10 µl) and its equivalent non-treated control were then diluted in 2.5 ml of 20 mM sodium acetate pH 4.5 buffer and analysed by EDC.

In some cases EDC was performed after fractionation of samples by size exclusion chromatography (SEC) which was performed using a 120 mL HiPrep 16/60 Sephacryl® S-400 HR column (GE Healthcare, 28-9356-04) which was eluted with 1 M NaCl in 20 mM sodium acetate buffer pH 4.5. 96 fractions of 1 mL were collected and 100 µl of each fraction were coated on microtitre plates for standard ELISA.

### 2.4 ELISA and sandwich ELISA

For ELISA screening of cell wall epitope occurrence in all fruits, each fraction i.e. water, CDTA, Na_2_CO_3_ and KOH extracts, was coated onto microtitre plates using 100 µl of the extracts diluted 60-fold in 1X PBS (phosphate-buffered saline: 137 mM NaCl, 2.7 mM KCl, 10 mM Na_2_HPO_4_, 2 mM KH_2_PO_4_) overnight at 4 °C. The ELISA analysis was performed following the protocol described in Cornuault et al., 2014. The analysis of each sample was performed in triplicate. For sandwich ELISAs microtitre plates were coated with the xylan-binding CBM2b1-2 (McCartney et al., 2006) overnight at 4 °C using 40 µg/ml in 1X PBS and the aubergine KOH extract was diluted 60-fold in 1X PBS with 5% (w/v) skimmed milk powder for the first stage incubation prior to standard ELISA.

## 3 Results

Four commercially important fruits were selected for cell wall analysis with the aim of providing variation in both taxonomic grouping and textural properties. These were tomato and aubergine (belonging to the Solanaceae family) and strawberry and apple (belonging to the Rosaceae family). Tomato and aubergine are berries for which the fleshy fruit parenchyma origin is the pericarp of ovule tissues; while both the Rosaceae species have false fruits. For strawberry, the fleshy fruit parenchyma is an enlarged flower receptacle, and for the apple it is the hypanthium – a fusion of petals, sepals and ovary walls. In all cases ripened, mature fruits were analysed. The texture analysis and cell wall content of these fruits can be found in Table 1 of Cornuault et al. (2017b) and indicated that tomato and strawberry have the softest textures, and that strawberry and apple have the higher % Brix (a ripening related parameter) that correlates with a sweeter taste of these in contrast to fruits considered vegetables like tomato and aubergine. Is noteworthy to comment how the firmer fruit depends on the textural parameter, thus, aubergine possess the highest hardness value, while apple has the highest elastic modulus. Additionally, the stress–strain curves (Fig. 1 of Cornuault et al., 2017b) graphically reflects the diverse textural features of each fruit species: plasticity of aubergine, crispness of apple, and softness of tomato and strawberry.

It should be noted that aubergine yielded the highest percentage of CWM and that strawberry had the highest level of highly soluble solids in the phenol extract. Equivalent weights of AIR were then sequentially extracted with equivalent volumes of water, the cation-chelator CDTA, Na_2_CO_3_ and KOH to provide four fractions of solubilized polymers for each species. These equivalent fractions were then used for immunoassays with sets of mAbs to pectic HG, pectic RG-I, xyloglucan and heteroxylan epitopes. The ELISA signals from equivalent dilutions of the extracts can be found elsewhere (Fig. 2, Cornuault et al., 2017b). The notable features of this analysis are that the esterified HG epitope (JIM7) was abundant in all four fruits in water and CDTA extracts. LM19 (unmethylesterified HG) was found in all fractions and especially after alkali treatment when all HG is demethylesterified. In terms of RG-I epitopes, the INRA-RU1 epitope was abundant in all fractions. However, the arabinan epitope (LM6-M) was prominently detected in strawberry and tomato whereas galactan (LM5) was the dominant side chain epitope in aubergine. Xyloglucan epitopes were most abundant in the water and KOH fractions of all four fruits. However, the LM15 epitope was strongly detected in apple and strawberry water extracts but much less, if not absent, in aubergine and tomato. Heteroxylan epitopes (LM11 and LM28) were specifically detected in the water and KOH fractions of aubergine cell walls. This primary analysis provided a guide to which mAbs use in later EDC analysis.

### 3.1 Spectra of pectic HG and RG-I molecules are abundant in the soluble fractions of tomato and strawberry cell wall preparations

**Fig. 1:**
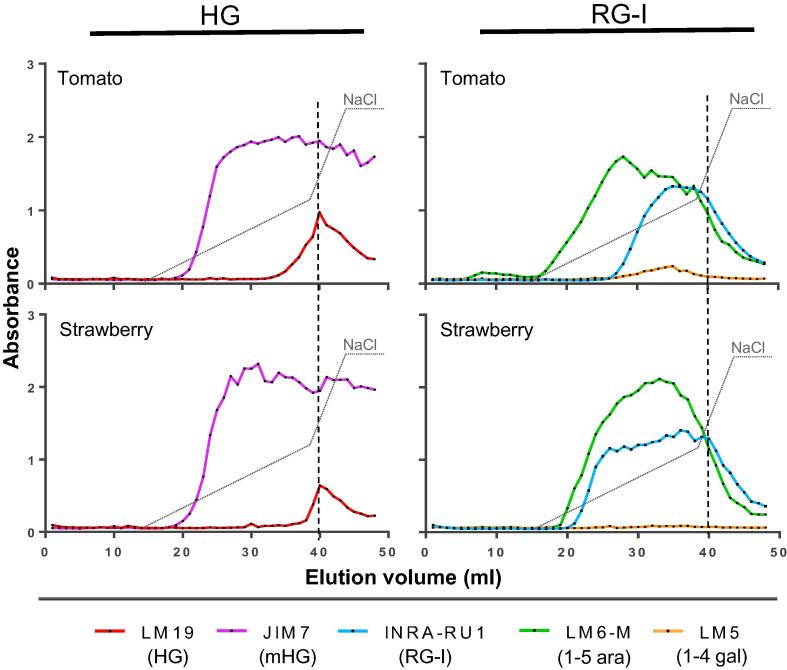
Epitope detection chromatography (EDC) profiles of pectic HG and RG-I epitopes in phenol-extracted highly soluble cell wall materials from tomato and strawberry. Co-elution profiles of no ester HG (LM19) and Me-ester HG (mHG) (JIM7) and RG-I backbone (INRA-RU1), arabinan (LM6-M) and galactan (LM5). Anion-exchange chromatography (AEC) with elution gradient (dotted line) from 0 to 0.3 M and from 0.3 to 0.6 M of NaCl. Averaged profiles from two biological replicates. LM19 epitope peaks are marked by vertical dashed grey lines.

**Fig. 2:**
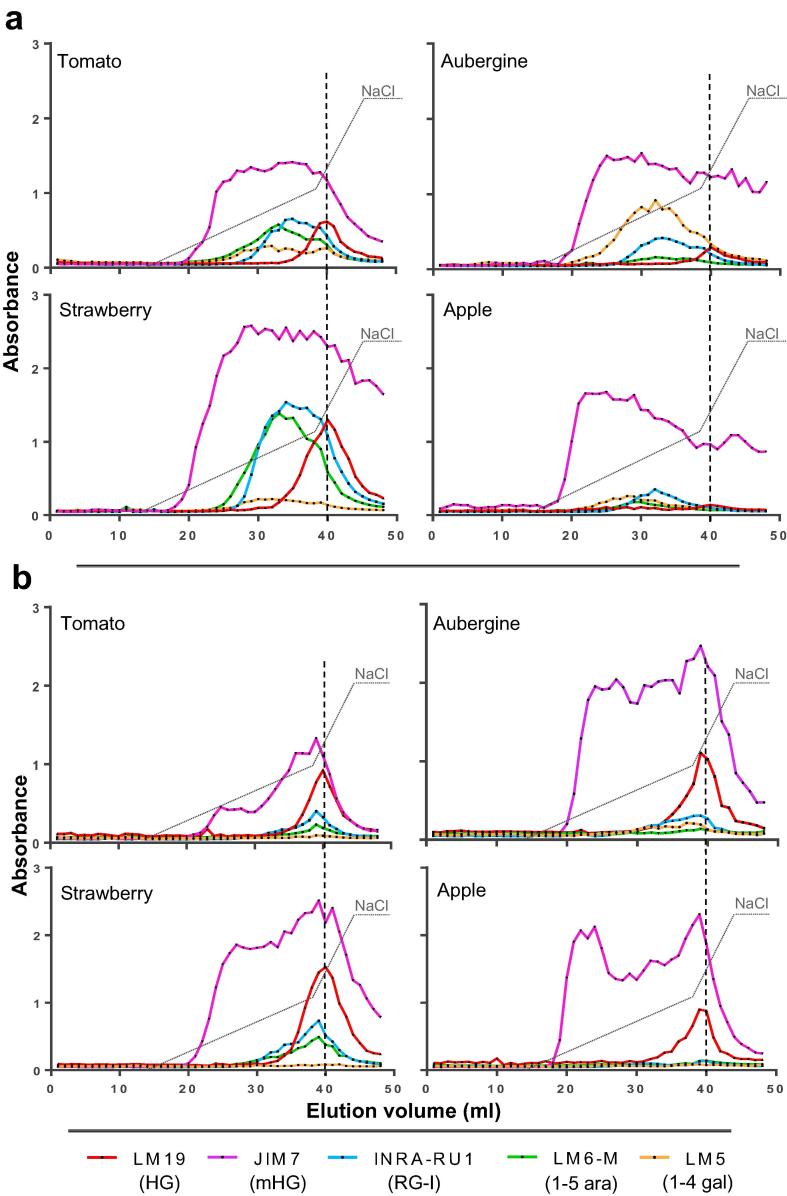
AE-EDC profiles of water- and CDTA-soluble pectins from tomato, aubergine, strawberry and apple fruits. a) EDC profiles HG and RG-I epitopes in water soluble fractions b) EDC profiles of HG and RG-I epitopes in CDTA-soluble fractions. In both cases AEC with elution gradient (dotted line) from 0 to 0.3 M and from 0.3 to 0.6 M of NaCl. Averaged profiles from two biological replicates. LM19 epitope peaks are marked by vertical dashed grey lines.

During the investigation it became apparent that substantial amounts of matrix polysaccharide epitopes were extracted at the phenol step and that this was particularly the case for tomato and strawberry (Fig. 1). Virtually no polysaccharides were extracted by phenol in apple and aubergine. The materials collected during the phenol extract were analysed by anion-exchange EDC (AE-EDC). The JIM7 methylester HG epitope was the dominant HG epitope in these highly soluble materials and JIM7-reactive material eluted at the onset of the salt elution gradient from 18 ml elution volume onwards and a spectrum of molecules eluting over a range of salt concentrations was detected (Fig. 1). It is to be noted here that the LM19 unesterified HG epitope was not distributed across the same range but detected in a discrete peak centered at 40 ml elution volume (Fig. 1). This indicates that the relatively small proportion of low esterified HG was separable from the main bulk of methylesterified HG and was mostly contained within a distinct set of more acidic molecules. Analysis of the RG-I related epitopes INRA-RU1, LM5 and LM6-M, in the same fractions, indicated strong signals in different regions of the gradient that did not co-elute precisely with the JIM7 methylesterified HG nor the LM19 unesterified HG epitopes (Fig. 1). In the tomato fraction the LM6-M arabinan epitope eluted early and ahead of the INRA-RU1 epitope which coincided with a low LM5 galactan signal and all epitopes peaking in abundance ahead of the unesterified HG LM19 signal peak. In strawberry the elution of the arabinan epitope was later than in tomato and was only marginally ahead of the INRA-RU1 signal. Equivalent AE-EDC analyses of water-soluble pectins, shown in Fig. 2a, reveal the same trends in epitope elution patterns again indicative of wide range of pectic molecules being present with the early elution of the dominant JIM7 epitope and late elution of the LM19 unesterified HG epitope and with the RG-I epitopes being intermediate.

### 3.2 CDTA-soluble pectins have a relatively higher proportion of unesterified HG and lower levels of RG-I epitopes

Equivalent analysis of CDTA-soluble pectins showed that these fractions also contained similar spectra of HG molecules with the firmer fruits, aubergine and apple, having methylester-rich HG eluting at the onset of the salt gradient (Fig. 2b). In the case of strawberry and, particularly tomato, the methylester-rich HG material did not elute quite as early and was less abundant. In the case of apple there was a striking two signal maxima for the JIM7 epitope at around 25 ml elution volume and one coincident with the unesterified HG peak. As the molecules were solubilized with a cation chelator it could be assumed that the earlier-eluting peaks/materials were calcium ion crosslinked and contained unesterified regions below the level of detection in these fractions or that the release of the LM19 epitope-rich HG resulted in an associated release of a methylester-rich-HG fraction by un-entanglement or other association. In all cases the RG-I associated signals were relatively low in these fractions with the RG-I epitopes broadly co-eluting with the un-esterified HG LM19 epitope peak although with detection of some earlier eluting materials.

### 3.3 Na_2_CO_3_ solubilizes pectic molecules rich in RG-I motifs

**Fig. 3:**
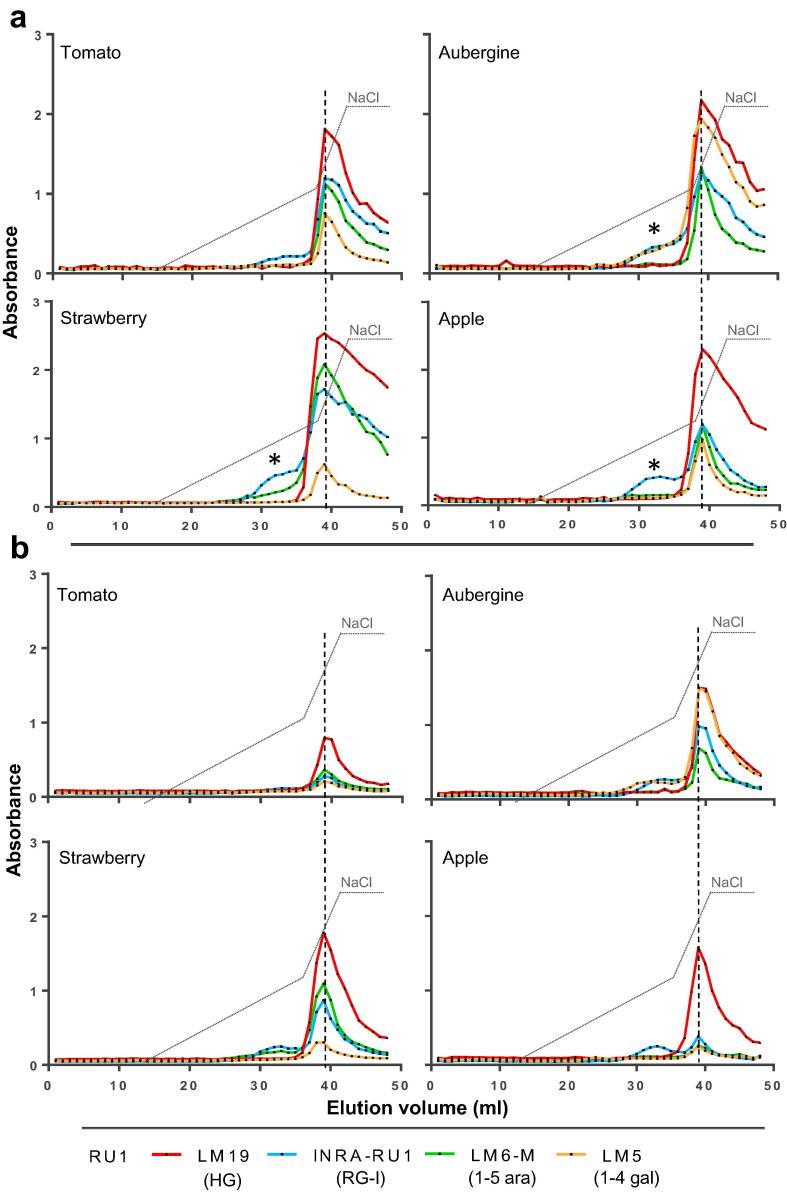
AE-EDC profiles of alkali-soluble pectins from tomato, aubergine, strawberry and apple fruits. a) EDC profiles of HG and RG-I epitopes in sodium carbonate-soluble fractions. b) EDC profiles of HG and RG-I epitopes in KOH-soluble fractions. AEC with elution gradient (dotted line) from 0 to 0.3 M and from 0.3 to 0.6 M of NaCl. Averaged profiles from two biological replicates. LM19 epitope peaks are marked by vertical dashed grey lines. ^*^ in a) indicate fractions of RG-I that may not be linked to HG.

After release of chelator-soluble molecules the AIRs were extracted with Na_2_CO_3_ and, as anticipated, no JIM7 epitope was detected in the solubilized fractions and the eluted HG, identified by LM19, was restricted to a peak at 40 ml elution volume upon AE-EDC analysis (Fig. 3a). Marking a clear difference with the chelator-soluble fractions, RG-I epitopes were abundantly present and mostly co-eluted with the LM19 signal peaks. In most cases, however, there was some indication of early elution shoulders for the RG-I epitopes. The materials extracted with KOH were similar in terms of EDC profile to the Na_2_CO_3_-extracted material although pectin epitopes were less abundant (Fig. 3b).

### 3.4 Subsets of RG-I motifs can be separated from HG domains

**Fig. 4:**
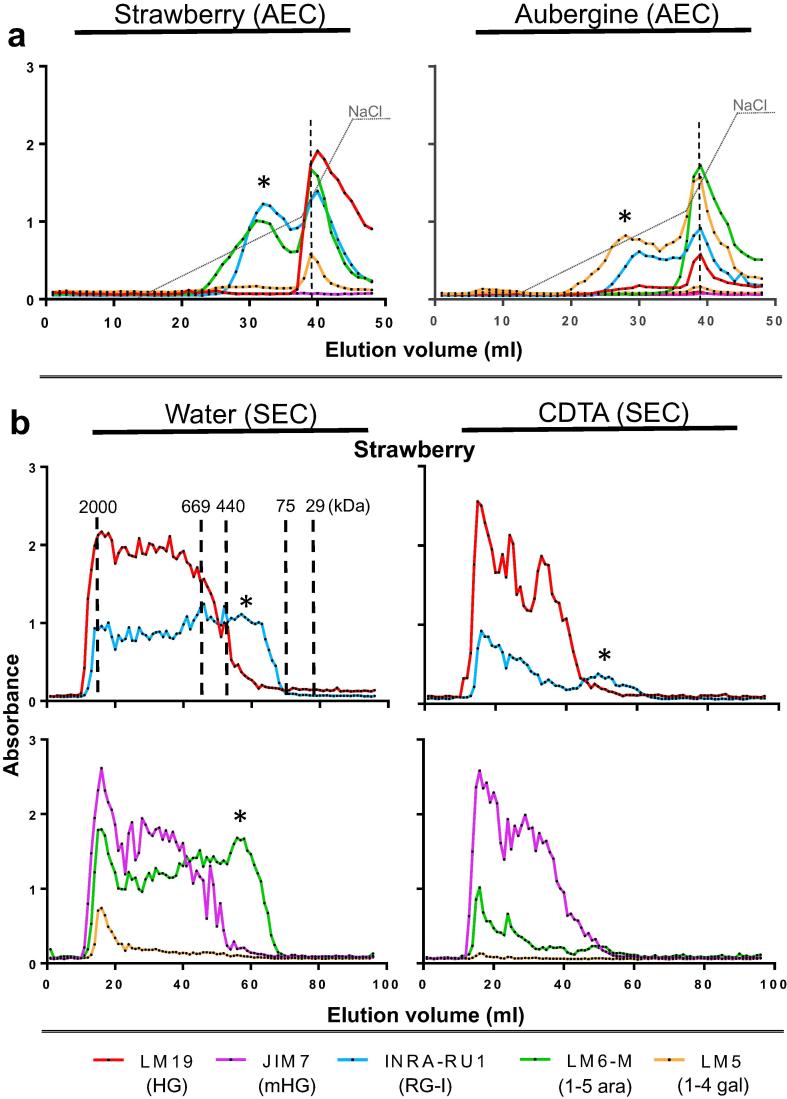
EDC profiles of strawberry and aubergine from water and CDTA extracts after AEC and size exclusion chromatography (SEC). a) Equivalent EDC for strawberry and aubergine water soluble samples after de-esterification treatment with 0.5 M Na_2_CO_3_ and 1% NaBH_4_, 2 h at RT. AEC with elution gradient (dotted line) from 0 to 0.3 M and from 0.3 to 0.6 M of NaCl. LM19 epitope peaks are marked by vertical dashed grey lines. b) Profile obtained by size exclusion EDC of untreated water and CDTA fractions from strawberry fruit. Signals obtained from same fractions are plotted in two graphs for extra-clarity. ^*^ indicates fractions containing RG-I but no detectable HG. The sizes indicated on the graph were obtained using the elution of proteins and polysaccharide of reference: blue dextran (2 MDa), thyroglobulin (669 KDa), ferritin (440 kDa), conalbumin (75 kDA) and carbonic anhydrase (29 kDa).

In the Na_2_CO_3_ fractions, particularly of aubergine and strawberry, there were early shoulders of RG-I epitopes eluting ahead of the main unesterified HG domains (Fig. 3a) indicating that some RG-I molecules in these fractions are separable from HG. The abundant RG-I epitopes eluting ahead of the unesterified HG peak in water-soluble and CDTA-soluble fractions (Fig. 4), could indicate attachment to methylester-rich domains or again the occurrence of separate RG-I molecules unattached to HG. This was explored further in the aubergine and strawberry fractions where RG-I epitopes were relatively abundant. Firstly, equivalent water-soluble samples were alkali-treated (to remove HG methylesters) prior to injection and AE-EDC analysis (Fig. 4a). In both strawberry and aubergine, in the absence of any detectable JIM7 HG epitope (i.e. HG is unesterified), the elution of the RG-I epitopes resolved into two peaks one of which co-eluted with the unesterified HG and one which eluted earlier indicating that it is separate from HG domains (asterisks in Fig. 4a). The strawberry water and CDTA fractions were also studied using EDC in a size-exclusion mode (SE-EDC) as shown in Fig. 4b. In this case, separation is based on size with larger polymers eluting first and smaller molecules eluting in later fractions. The SE-EDC epitope profiles indicated that the detection of the INRA-RU1 and the LM6-M epitopes in a late-eluting region, after HG epitopes, confirming the presence of smaller RG-I molecules unattached to HG. This was particularly clear in the water fraction (Fig. 4b).

### 3.5 Xyloglucan epitopes are differentially associated with pectic HG molecules in alkali-soluble fractions

**Fig. 5:**
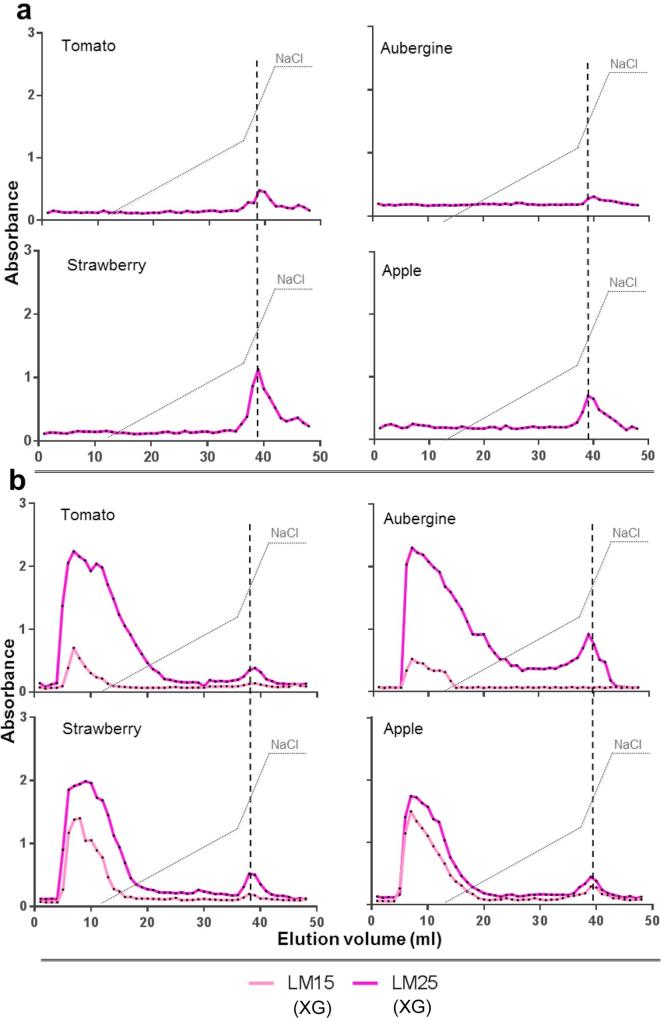
EDC profiles of xyloglucan epitopes in alkali-soluble fractions. AEC with elution gradient (dotted line) from 0 to 0.3 M and from 0.3 to 0.6 M of NaCl. Averaged profiles from two biological replicates. a) LM25 epitope profiles in Na_2_CO_3_-soluble fractions of tomato, aubergine, strawberry and apple. Position of LM19 HG peak (Fig. 4a) is shown by vertical grey dashed line. b) LM25 and LM15 XG EDC profiles in KOH-soluble fractions of tomato, aubergine, strawberry and apple. Position of LM19 epitope peaks (Fig. 4b) are marked by a vertical grey dashed line.

Xyloglucan is the major non-pectic matrix polysaccharide in primary cell walls of the eudicots that includes the Solanaceae and Rosaceae. Analysis of two XG epitopes (LM15 and LM25) in all fractions (water, CDTA, Na_2_CO_3_ and KOH) indicated that XG was widely detected (see Fig. 2 in Cornuault et al., 2017b). The LM25 epitope was detected to some extent in the Na_2_CO_3_ fractions for all four species exclusively co-eluting with pectic HG (Fig. 5a). As expected the xyloglucan epitopes were most abundant in the KOH fractions and here in addition to a large neutral peak there was also for all four fruits some co-elution with the pectic HG peak and this was most significant for the aubergine KOH extract (Fig. 5b). The low LM15 signals, relative to the LM25 signals, in the tomato and aubergine (Fig. 5b) likely reflects the occurrence of the specific arabinoxyloglucan structure present in the Solanaceae (Hoffman et al., 2005).

### 3.6 Two populations of heteroxylan polysaccharides occur in aubergine cell walls and some is associated with xyloglucan and pectic molecules

**Fig. 6:**
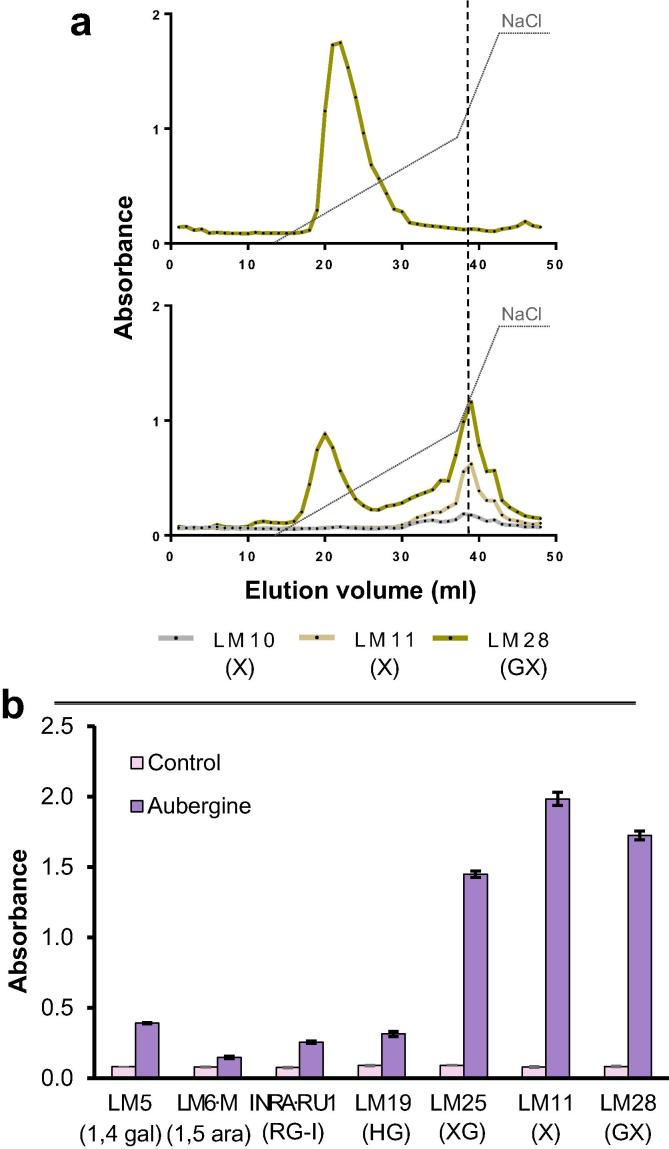
Heteroxylan epitopes in aubergine fruits. a) EDC profiles of heteroxylan epitopes in aubergine parenchyma cell walls in water-soluble fraction and KOH-soluble fraction. The vertical dotted line marks the elution volume of the LM19 HG epitope. AEC with elution gradient (grey dotted line) from 0 to 0.3 M and from 0.3 to 0.6 M of NaCl. Averaged profiles from two biological replicates. b) Sandwich ELISA using CBM2b,1-2 as capture probe against polysaccharides of KOH 4 M fraction from aubergine. Pectin (LM5, LM6, INRA-RU1, LM19), xyloglucan (LM25) and heteroxylan probes (LM11, LM28) were used to detect potential interlinkages between polysaccharide classes. Values displayed are means of 3 technical replicates with SE shown by error bars.

Heteroxylans are a group of cell wall polysaccharides found in abundance in the secondary cell walls of eudicot plants. Aubergine cell walls, unlike those of the other three fruits contain abundant amounts of heteroxylan epitopes in the water and KOH fractions, particularly of the LM28 glucuronoxylan (GX) epitope (see Fig. 1 in Cornuault et al., 2017b). The presence of the LM28 epitope in the water fraction indicates part of the GX population was not tightly linked in to the cell wall. AE-EDC analysis indicated that in the water fraction the LM28 epitope eluted in a clear peak at the onset of the salt gradient (Fig. 6a). The AE-EDC profile of the KOH fraction indicated that the LM28 epitope eluted at a similar, but slightly earlier position and that also an approximately equivalent amount was detected in a second peak that co-eluted with the xylan epitopes LM10 and LM11 (not detectable at the first-eluting peak) and also the LM19 HG epitope (Fig. 6a). Sandwich ELISA analysis using xylan-binding CBM2b1-2 (McCartney et al., 2006) as the capture molecule and incubation with the KOH-fraction indicated signals for xylan mAbs, and also to some extent pectic epitopes and a very strong signal for xyloglucan (Fig. 6b) suggesting that the xylan polysaccharide was linked to pectin and xyloglucan.

## 4 Discussion

Cell wall remodelling is a major factor underpinning fruit texture changes during ripening and several genetic transformation studies support a crucial role for polysaccharides in fruit firmness and shelf life (Toivonen & Brummell, 2008). The aim of this work was to apply new methodological approach to understand aspects of cell wall architecture and its relevance on final fruit textures. Cell wall fractionation in several solvents of increasing stringency was the chosen methodology to enable the analysis of isolated cell wall polysaccharides. To ensure that our analyses were of native polysaccharides we included phenol in the first step to prevent sample degradation by endogenous cell wall enzymes, and reducing agent and low temperatures during alkali extraction steps to prevent alkali peeling of polysaccharides.

A major insight from this work relates to the heterogeneity of detected HG polymers in that acidic pectic molecules (tagged by the LM19 epitope) are to some extent separable from methylester-rich HG (tagged by JIM7 epitope). Additionally, in the analysis of these materials RG-I epitopes are not always associated with HG. Moreover, in each fruit a broadly similar proportion of xyloglucan co-elutes with acidic pectic molecules. We propose these to be baseline profiles of matrix glycan heterogeneity of relevance to primary cell walls of many parenchyma-like tissues. One distinctive feature is the presence of abundant heteroxylan epitopes in aubergine fruit cell walls and not detected in the other fruits.

### 4.1 mAb-based approaches to cell wall analyses and the heterogeneity of pectic molecules

mAbs are powerful tools for the *in situ* detection of epitopes in the contexts of intact cell walls, cells, tissues and organs. Although caution must be observed in interpretations in that a mAb may not always be an absolute marker for a polymer class and the absence of an epitope is not a demonstration of the absence of a polymer. For example, the JIM7 HG mAb does not bind if HG is fully unesterified (Clausen et al., 2003). Other subtle modifications could possibly lead to loss of an epitope whilst the polymer remains present. For example, the LM5 galactan antibody has recently been shown to bind to non-reducing termini (Andersen et al., 2016) and a terminal modification may lead to epitope but not polymer loss in some contexts. These assessments are limited to some extent by our knowledge of probe specificity and also of course probe availability. The absence of an available monoclonal antibody to RG-II is a particularly pertinent gap for understanding the structure of pectic molecules using these approaches. However, even if not all desired probes are available the mAbs used have a robust history of use and are demonstrated markers of cell wall matrix polysaccharides and we show here that this approach is useful for the rapid dissection of polysaccharide heterogeneity and potential interlinkages.

Here we have observed the heterogeneity of HG in relation to unesterified and acidic domains and that most RG-I is associated with unesterified HG. The identification of substantial amounts of RG-I molecules with no detectable association to a HG domain could indicate that RG-I polymers are deposited in cell walls on their own or are cleaved from larger complex pectic molecules (including both RG-I and HG) during cell wall re-modelling. Regarding RG-I, the diversity of this domain is reflected by differences in the occurrence of its side chains with LM5 (galactan) and LM6-M (arabinan) epitopes. While arabinan is the predominant RG-I side chain in the phenol and water extracts (Fig. 1, Fig. 2 respectively) of tomato and strawberry; galactan (LM5) is the most detected neutral side chain in almost all fractions in aubergine. Moreover, EDC profiles also indicate a pool of arabinan (LM6-M signal) independent from the backbone RG-I (INRA-RU1) epitope in tomato, that was not detected in strawberry or phenol extracts (Fig. 1). How the heterogeneities identified here reflect metabolic processes, perhaps synthesis or *in muro* metabolism, is yet to be determined.

### 4.2 Interactions and associations between sets of cell wall matrix polysaccharides

Covalent links between pectin and xyloglucan are well documented (Park & Cosgrove, 2015) and the recent identification of links between arabinoxylan, AGP and pectin in APAP1 (Tan et al., 2013) indicate that matrix-glycan inter-linkages are likely to be an important feature of cell walls. The XG profiles obtained for all four fruits are broadly similar and suggest that all of them possess a sub-population of pectin attached to XG. This fraction is in part released by Na_2_CO_3_. The bulk of XG is released from the wall during the KOH treatment, mostly being neutral, but a small component of this also co-elutes with pectin (Fig. 5). It may be that this subset of pectin is linked to a subset of xyloglucan to ensure tight attachment to cellulose microfibril surfaces through hydrogen bonding. This may be part of a mechanism linking pectin through it supramolecular assemblies to contribute to cell wall cohesion and strength. Through the use of LM25 and LM15 xyloglucan mAbs we can observe the difference in XG substitution existing between the Rosaceae and the Solanaceae (a lower relative level of the XXXG xyloglucan epitope of LM15 in tomato/aubergine) – a difference that is the only obvious pattern in the work reported here that is potentially associable with phylogeny (Fig. 5b).

The case of heteroxylan in aubergine is particularly interesting. Again the significance of a water-soluble glucuronoxylan (GX) and presence of potentially related polymer although with different elution characteristics in the KOH fraction leads to questions of metabolic relationships of these heteroxylans (Fig. 6a). This may reflect aspects of cell wall deposition and assembly or re-modelling of the cell wall during fruit development. It is of particular interest here that xyloglucan appears to be part of this pectin-heteroxylan interlinkage (Fig. 6b) and it will be of interest to elucidate the specific arrangement of pectin, xyloglucan and xylan domains. A similar supramolecule of pectin-xylan-xyloglucan has been described in cauliflower stem (Femenia et al., 1999). These potential interactions now require detailed structural characterisation and are likely to be key to achieving a better understanding of cell wall architectures and cell wall and organ properties.

### 4.3 Profiles of cell wall matrix polysaccharide and possible relationships with fruit mechanical properties

Texture is a multifaceted feature and an adequate selection of parameters is crucial, especially when comparing fruits of such diverse and contrasting texture (Fig. 1, Cornuault et al., 2017b). In our study, aubergine possess the highest value of hardness, however apple is the stiffer fruit according to the elastic modulus (E) (Table 1, Cornuault et al., 2017b). Hardness, defined as maximum force, has little biological meaning but is widely used in the food industry because it is easy to obtain; by constrast, the elastic modulus (E) is a rigorous measurement of sample stiffness, but because is not a straightforward measurement (the slope in the elastic zone), its use is not so widespread. Within the four chosen fruits tomato and strawberry displayed the lowest values of hardness and elastic modulus (Table 1, Cornuault et al., 2017b) with stress–strain curves that present inflection points instead of prominent peaks, denoting their soft texture as a main feature.

A noticeable difference between tomato and strawberry (soft) fruits and aubergine and apple (firmer) was the higher extractability of subsets of their cell wall pectins. This pectin solubilization is well documented as a main event during softening of fleshy fruits (Redgwell et al., 1997, Toivonen and Brummell, 2008). Both fruits released larger amounts of pectin to the phenol extract that were mostly composed of highly methylesterified HG and arabinan-rich RG-I (Fig. 1). As these fruits were used at point of consumer use, as was confirmed by the % Brix values for each fruit (Table 1, Cornuault et al., 2017b), ripening mechanisms would be underway. Fruit ripening has been shown to involve the action of cell wall modifying enzymes primarily targeting pectin and hemicelluloses (Toivonen & Brummell, 2008). The high levels of soluble methylesterified HG might therefore be the product of the degradation of regions of de-esterified HG releasing relatively large amounts of methylesterified HG and RG-I. Strawberry and tomato in particular both possess high levels of soluble esterified HG which may relate to lower tissue firmness. Results reported here may suggest that in strawberry and tomato, two fruits with soft texture, the de-esterification of HG results in small stretches of HG for polygalacturonases to digest with a consequence of highly methylesterified HG “in solution” in the cell wall, causing the swelling of the cell wall, a loss of firmness of the tissue and the disruption of cell adhesion, a complex scenario well described by Sénéchal, Wattier, Rustérucci, & Pelloux, 2014. In the case of aubergine, texture is depicted by a typical stress–strain curve with only one sharp peak at maximum strain (70%) (Fig. 1 in Cornuault et al., 2017b). The higher cell wall content could lead to its ductile behavior under stress, with thick walls resisting high stress levels by plastic deformations. Aubergine also showed a higher pectin signal in the KOH fraction (Fig. 2 in Cornuault et al., 2017b) – which is likely due to association with xylan/xyloglucan domains – contributing to a more tightly linked and firmer cell wall structure, able to develop a firmer fruit texture with a ductile behavior at tissue-organ level.

One driver of this work was to consider arabinan- and galactan-rich forms of RG-I. Here we have looked at galactan and arabinan epitopes as indicators of the presence of neutral RG-I side chains. The function of arabinan and galactan side chains in cell wall mechanical properties is still unclear. Overall, in this study the prevalence of galactan or arabinan, unlike XG decoration, doesn’t seem to correlate with the fruit phylogeny. There is an indication that the firmer fruits possess relatively higher levels of galactan and it is notable that the LM5 galactan epitope is relatively abundant in the KOH fractions of aubergine and apple indicating that a sub-population of RG-I is tightly linked into cell walls – possibly contributing to the mechanical properties of these firmer fruits.

Consistently lower signals were observed for RG-I in apple extracts compared to the other fruits. This is in agreement with the low pectin solubilization and the high proportion of arabinose and galactose that remains tightly bound to the wall even after KOH extraction (Redgwell et al., 1997). Loss of branched arabinan side chains of RG-I have being correlated with a reduction of fruit firmness during postharvest in apple (Peña & Carpita, 2004). Evidence has indicated the importance of a greater connective integrity of apple cell wall architecture (Redgwell, Curti, & Gehin-Delval, 2008). In this context, direct interaction between pectin and cellulose could be crucial and linkages between these polymers has been reported (Wang and Hong, 2016, Zykwinska et al., 2005). The low signals for RG-I side chain epitopes in apple could be due to a recalcitrant subset of RG-I linked to cellulose microfibrils. Further analyses are necessary to confirm this. This could explain the difference in texture between apple, a fruit characterized by a crisp texture which has almost no elasticity but instead collapses at breaking point followed by subsequent jagged peaks (see Fig. 1 in Cornuault et al., 2017b), and the two softer fruits. Tomato and strawberry release unesterified HG in water extractions whereas aubergine and apple release it in CDTA or later extraction steps. This variation in water vs CDTA extractability may reflect the variation in calcium ion content of the cell wall of the different fruits. It has been reported that calcium-ion treated strawberry preserved cell wall integrity for longer during post-harvest (Lara, Garcia, & Vendrell, 2004). This study also indicated that calcium-treated fruit released less unesterified pectin in water than the untreated one. Therefore the concentration of cell wall calcium is certain to play an important role in maintaining cell adhesion and influence fruit firmness through its interaction with pectic networks.

Aubergine cell wall studies are scarce and few data are available (Esteban et al., 1993, Zaro et al., 2014). In our study, aubergine has the highest cell wall yield that likely reflects the continuous cell wall deposition during fruit ontogeny (Esteban et al., 1993, Zaro et al., 2014), and the lack of or reduced cell wall degradation that is a characteristic feature during ripening in most fruits (Brummell, 2006). Aubergine is the only fruit of the four analysed here to have a heteroxylan component and the galactan-rich RG-I appears tightly linked within the cell walls suggestive of a highly cross-linked wall matrix. Apple has also been shown to have a pectin-rich KOH fraction and this strongly bound pectin is correlated with firmer fruit texture (Redgwell et al., 2008).

Overall, this work presents high throughput methodologies to determine pectic and matrix polysaccharides sub-populations and extends our understanding of the existence of interlinkages between polymer classes. These potential interactions are likely to be a dynamic aspect of cell wall architectures that may be key to achieve a better understanding of cell wall structures, their re-modelling and organ mechanical properties.
